# Magnetic dynamics of hedgehog in icosahedral quasicrystal

**DOI:** 10.1038/s41598-022-19870-6

**Published:** 2022-09-15

**Authors:** Shinji Watanabe

**Affiliations:** Department of Basic Sciences, Kyushu Institute of Technology, Kitakyushu, Fukuoka 804-8550 Japan

**Keywords:** Magnetic properties and materials, Physics, Condensed-matter physics, Topological matter, Topological defects

## Abstract

Quasicrystals (QCs) possess a unique lattice structure without translational invariance, which is characterized by the rotational symmetry forbidden in periodic crystals such as the 5-fold rotation. Recent discovery of the ferromagnetic (FM) long-range order in the terbium-based QC has brought about breakthrough but the magnetic structure and dynamics remain unresolved. Here, we reveal the dynamical as well as static structure of the FM hedgehog state in the icosahedral QC. The FM hedgehog is shown to be characterized by the triple-*Q* state in the reciprocal-lattice ${{\varvec{q}}}$ space. Dynamical structure factor is shown to exhibit highly structured ${{\varvec{q}}}$ and energy dependences. We find a unique magnetic excitation mode along the 5-fold direction exhibiting the streak fine structure in the ${{\varvec{q}}}$-energy plane, which is characteristic of the hedgehog in the icosahedral QC. Non-reciprocal magnetic excitations are shown to arise from the FM hedgehog order, which emerge in the vast extent of the ${{\varvec{q}}}$-energy plane.

Quasicrystal (QC) has a unique lattice structure with rotational symmetry forbidden in periodic crystals^1^. Although progress has been made in unraveling their atomic structure^2,3^, the understanding of their electric properties remains a challenging and fascinating problem, because the Bloch theorem can no longer be applied.

The unresolved vital issue has been whether the magnetic long-range order is realized in the three-dimensional QC^4–16^. Recently, the ferromagnetic (FM) long-range order has been discovered experimentally in the QC Au-Ga-Tb^17^. Theoretically, the FM long-range order has actually been shown to be realized in the QC Au-SM-Tb (SM=Si, Al, Ge, Sn and Ga)^18,19^. Interestingly, the hedgehog state, where the magnetic moments at the Tb site located at each vertex of the icosahedron (IC) is directed outward (see Fig. 1A), has been shown to form a uniform long-range order as shown in Fig. 1B^18^. Moreover, the hedgehog state on the IC has been revealed to be characterized by the topological invariant, i.e., the topological charge of one, which exhibits emergent phenomena such as the topological Hall effect^18^.

Although the FM order has been detected in the QC, the detailed magnetic structure has not been resolved experimentally^17^. Theoretically, the configurations of the magnetic moments in real space have been identified but their magnetic structure factor in reciprocal space has not been clarified^18,19^. Furthermore, the dynamical property of the magnetism in the QC remains unresolved.

As for the dynamics in the QC, the lattice dynamics was studied by inelastic X-ray and neutron scattering measurements^20,21^. The dynamical structure factor was theoretically calculated in the spin 1/2 Heisenberg model on the Fibonacci chain for the FM ground state^22^ and in two-dimensional systems^23^. The dynamical structure factor was also calculated for antiferromagnetic spin 1/2 Heisenberg model on the two-dimensional octagonal tiling^24^. However, little has been known about the magnetic dynamics in the real three-dimensional QC theoretically nor experimentally.

In this report, we present the dynamical property of the uniform long-range order of the hedgehog state in the Tb-based QC. By calculating the magnetic structure factor, we show that the hedgehog is characterized as the triple-*Q* state. By analyzing the dynamical structure factor, we reveal unique energy and momentum dependences of the magnetic excitations. We find that the magnetic excitation mode along the 5-fold axis direction exhibiting streak fine structure with periodicity characterized by the wavelength of the diameter of the IC, which is considered to be characteristic of the hedgehog in the icosahedral QC. We also find the non-reciprocal magnetic excitation mode in the QC. We note that we take the unit of $\hbar =1$ hereafter where $\hbar$ is reduced Planck constant.

**Figure 1:**
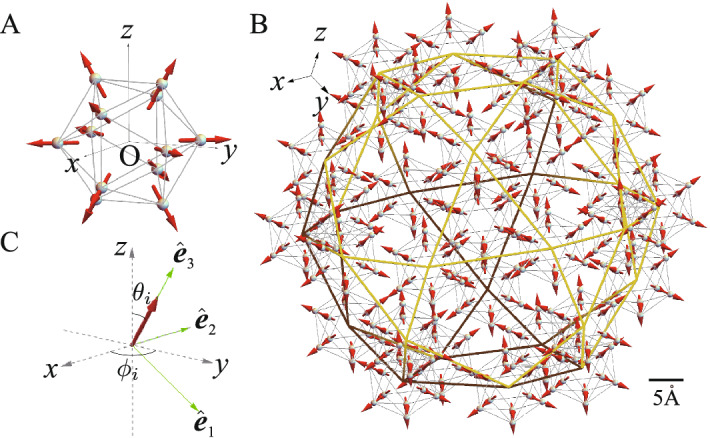
(**A**) The hedgehog state in the IC. Each arrow illustrates the magnetic moment at Tb, which is directed to the pseudo 5-fold axis. (**B**) The hedgehog state in Cd$_{5.7}$Yb-type QC. Green (brown) lines at the front (back) connect the vertices of the icosidodecahedron. Scale bar (5 Å) is shown in (**B**). (**C**) Local coordinate at the Tb site with the orthogonal unit vectors $\hat{{\varvec{e}}}_1$, $\hat{{\varvec{e}}}_2$, and $\hat{{\varvec{e}}}_3$ (see text).

## Results

### Lattice structure of QC

Let us start with the lattice structure of the QC. Although the FM long-range order has recently been identified by bulk measurements in the QC Au$_{65}$Ga$_{20}$Tb$_{15}$, the detailed lattice structure has not been solved experimentally^17^. In general, the rare-earth atoms in the rare-earth-based icosahedral QC are considered to form the lattice structure of Yb in the Cd$_{5.7}$Yb-type QC^3^. Figure 1B shows the main structure of the QC where the Tb-12 cluster, i.e., IC is located at each vertex of the icosidodecahedron with the total number of the vertices being 30. In the Cd$_{5.7}$Yb-type QC, there exists a few other ICs as well as Tb sites located between the ICs. In this study, as a first step of analysis, we consider the Tb sites shown in Fig. 1B with the total lattice number $N=12\times 30=360$ to get insight into the magnetic dynamics in the QC. Here, we employ the real Tb configuration for the IC (see Fig. 1A) as well as the icosidodecahedron in the 1/1 approximant crystal (AC) Au$_{70}$Si$_{17}$Tb$_{13}$ whose lattice structure was solved by the X-ray measurement^25^, as a typical case. The diameter of the IC is 10.56 Å. In Fig. 1B, the IC is located at 30 vertices of the $\tau ^3$-times enlarged icosidodecahedron in the Tsai-type cluster of Au$_{70}$Si$_{17}$Tb$_{13}$ with $\tau$ being the golden mean $\tau \equiv (1+\sqrt{5})/2$.

### Minimal model in rare earth-based QC

The Tb$^{3+}$ ion with $4f^8$ configuration has the ground state of the crystalline electric field (CEF) with the total angular momentum $J=6$ according to the Hund’s rule. The quantization axis of the CEF is the vector passing through each Tb site from the center of the IC, which is the pseudo 5-fold axis (see Fig. 1A). The detailed analysis of the CEF has revealed that the magnetic anisotropy arising from the CEF plays a key role in realizing the unique magnetic state such as the hedgehog on the IC^18,19^. Then, we consider the minimal model for the magnetism in the Tb-based QC as

1 $$\begin{aligned} H=\sum _{\langle i,j\rangle }J_{ij}{{\varvec{S}}}_i\cdot {{\varvec{S}}_j}-D\sum _{i}({{\varvec{S}}}_i\cdot \hat{{\varvec{e}}}_3)^2, \end{aligned}$$

where $J_{ij}$ is the exchange interaction between the *i*th and *j*th Tb sites and ${{\varvec{S}}}_i$ is the “spin” operator with $S_i=6$. In the second term, the unit vector $\hat{{\varvec{e}}}_3$ indicates the direction of the magnetic anisotropy arising from the CEF, which can be controlled by the compositions of Au and SM in Au-SM-Tb^18,19^. This model is expected to be relevant to not only the Tb-based QC but also rare-earth based QCs. In this study, to discuss the hedgehog state, $\hat{{\varvec{e}}}_3$ is set to be the pseudo 5-fold axis direction. For the strong limit of the magnetic anisotropy, it has been shown that the uniform long-range order of the hedgehog state is realized in the QC for $J_2/J_1>2$ where $J_1 (J_2)$ is the nearest neighbor (N.N.) (next N.N.) exchange interactions (Supplementary information, Fig. S1). Each IC is characterized by the topological charge of one $n_{\mathrm{TC}}=+1$, which is distributed quasi-periodically in Fig. 1B^18^. The hedgehog is the source of emergent field, which is regarded as monopole with the charge $n_{\mathrm{TC}}=+1$^26,27^.

**Figure 2:**
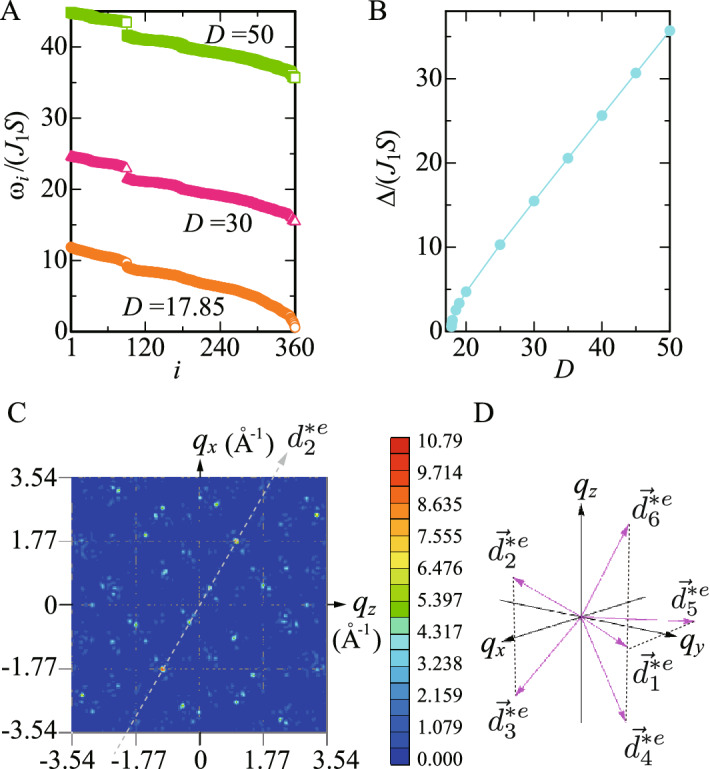
(**A**) $\omega _i$ vs *i* for $J_1=1.0$ and $J_2=2.3$ with various *D*. (**B**) CEF excitation gap vs *D*. (**C**) Top view of $F_{s}({{\varvec{q}}})$ in the $q_z$-$q_x$ plane for $q_y=0$. The gray dashed line in (**C**) denotes the pseudo 5-fold axis $d_2^{*e}$ defined in (**D**). (**D**) Primitive vectors in the six-dimensional reciprocal-lattice space ${\vec{d}}_i^{*e}$ $(i=1,\ldots , 6)$ as the physical (external) space components.

### Magnetic excitation in QC

In the hedgehog state, “spins” are non-collinearly aligned as shown in Fig. 1A. Hence it is convenient to introduce the local coordinate at each Tb site where the $\hat{e}_3$ axis is set as the ordered “spin” direction as shown in Fig. 1C (see Methods section for detail). Then, by applying the Holstein-Primakoff transformation^28^ to *H*, the “spin” operators are transformed to the boson operators as $S_i^{+}=\sqrt{2S-n_i}a_i$, $S_i^{-}=a_i^{\dagger }\sqrt{2S-n_i}$ and ${{\varvec{S}}}_i\cdot \hat{{\varvec{e}}}_3^i=S-n_i$ with $n_i\equiv a_i^{\dagger }a_i$. Here, $S_i^{-} (S_i^{+})$ is the lowering (raising) “spin” operator and $a_i (a_i^{\dagger })$ is an annihilation (creation) operator of the boson at the *i*th Tb site. Here the quadratic terms of the boson operators are retained because the higher order terms are considered to be irrelevant at least for the ground state.

We employ $J_1=1.0$ and $J_2=2.3$ as a typical parameter for the Tb-based QC. Actually, $J_2/J_1=2.3$ has been experimentally identified in the model Eq. (1) for the large *D* limit applied to the 1/1 AC Au$_{72}$Al$_{14}$Tb$_{14}$^29^. We confirmed that the hedgehog state shown in Fig. 1B with the $N=360$ sites under open boundary condition is realized as the ground state for $D\ge 17.85$ in Eq. (1), which gives the positive excitation energy $\omega _i$ for $i=1, \cdots , N$, as shown in Fig. 2A. The *D* dependence of the lowest excitation energy, i.e., the gap $\Delta \equiv \omega _N/(J_1S)$ between the first-excited energy and the ground enegy is shown in Fig. 2B. In the spectrum, there exist several gaps, as remarkably seen in Fig. 2A as $\Delta _1\equiv (\omega _{90}-\omega _{91})/(J_1S)$. As *D* increases, the energy gap $\Delta$ as well as $\Delta _1$ increases. Hereafter, we show the results for $D=30$ as the representative case. The lowest and highest energies of the excitation spectrum are $\Delta =\omega _N/(J_1S)= 15.47$ and $\omega _1/(J_1S)=24.59$, respectively.

### Static structure factor of magnetism

Then we calculate the magnetic structure factor

2 $$\begin{aligned} F_{s}({{\varvec{q}}})=\left\langle \left| \frac{1}{N}\sum _{i}{{\varvec{S}}}_{i}e^{i{{\varvec{q}}}\cdot {{\varvec{r}}}_i}\right| ^2 \right\rangle . \end{aligned}$$

The largest peak is located at ${{\varvec{Q}}}_1\equiv (1.77,0,1.02)~{\text{\AA} }^{-1}$ as shown in Fig. 2C. Since the alignment of the magnetic moments in the hedgehog shown in Fig. 1A is invariant under the permutation of *x*, *y*, and *z* axis, the same results in $F_s({{\varvec{q}}})$ as Fig. 2C are obtained by replacing $(q_x,q_y,q_z)$ with $(q_y,q_z,q_x)$ and also with $(q_z,q_x,q_y)$. Indeed we confirmed that the largest peak in $F_s({{\varvec{q}}})$ appears at ${{\varvec{Q}}}_2\equiv (0,1.02,1.77)$ and ${{\varvec{Q}}}_3\equiv (1.02,1.77,0)$ in $F_s({{\varvec{q}}})$ (Supplementary information, Figs. S2A and S2B). Namely, $F_s({{\varvec{Q}}}_1)=F_s({{\varvec{Q}}}_2)=F_s({{\varvec{Q}}}_3)$ holds. Thus the hedgehog state is characterized by the triple-*Q* $({{\varvec{Q}}}_1, {{\varvec{Q}}}_2$, and ${{\varvec{Q}}}_3)$ state.

In Fig. 2C, the spots lie along the pseudo 5-fold axis indicated by the dashed line with an arrow named $d_i^{*e}$. Here, ${\vec {d}}_i^{*e}$ $(i=1, \cdots , 6)$ is the primitive vector of the six-dimensional reciprocal lattice space as the physical (external) space components as shown in Fig. 2C^30^. Hereafter, we express the pseudo-5 fold axis for the ${\vec {d}}_i^{*e}$ direction as the $d_i^{*e}$ line with an arrow. We note that the slope of the $d_i^{*e}$ line for $i=2$ in Fig. 2C is 1.736 reflecting the real configuration of the Tb sites in the IC^25^ shown in Fig. 1A, which is known to be $\tau$ in the regular IC^30^. The slope of the $d_3^{*e}$, $d_5^{*e}$, and $d_4^{*e}$ lines is the sign-reversed value of the slope of the $d_2^{*e}$, $d_1^{*e}$, and $d_6^{*e}$ lines within each $q_z$-$q_x$, $q_x$-$q_y$, and $q_y$-$q_z$ plane, respectively (see Fig. 2D).

It is noted that $S_{xx}({{\varvec{q}}})$, $S_{zz}({{\varvec{q}}})$, and $S_{yy}({{\varvec{q}}})$ have the maximum at ${{\varvec{q}}}={{\varvec{Q}}}_1$, ${{\varvec{Q}}}_2$, and ${{\varvec{Q}}}_3$, respectively, where $S_{\alpha \beta }({{\varvec{q}}})$ is defined as $S_{\alpha \beta }({{\varvec{q}}})\equiv \frac{1}{N}\sum _{i,j}e^{i{{\varvec{q}}}\cdot ({{\varvec{r}}}_i-{{\varvec{r}}}_j)}\langle S_{i\alpha }S_{j\beta }\rangle$ $(\alpha =x, y,$ and *z*).

**Figure 3:**
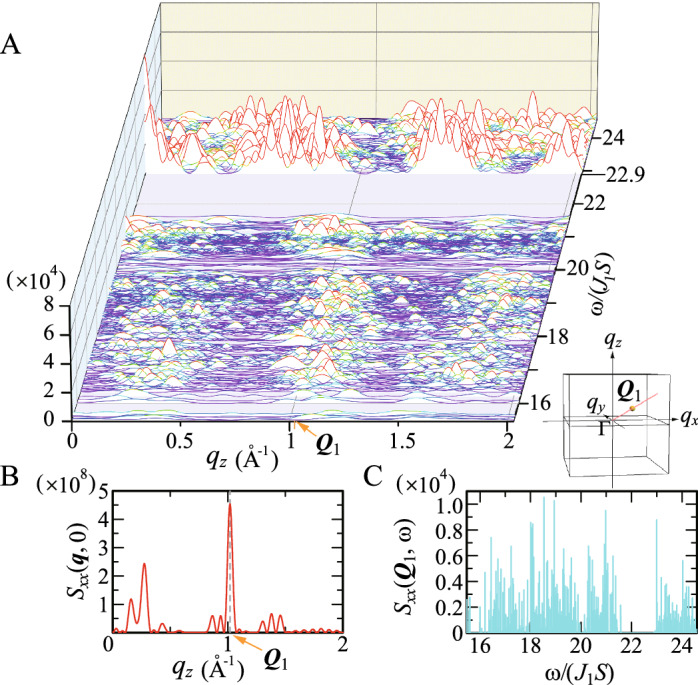
Dynamical structure factors (**A**) $S_{xx}({{\varvec{q}}},\omega )$ and (**B**) $S_{xx}({{\varvec{q}}},0)$ for ${{\varvec{q}}}$ along the $d_2^{*e}$ line through ${{\varvec{Q}}}_1$ with $q_y=0$. Inset illustrates the $d_2^{*e}$ line through ${{\varvec{Q}}}_1$ inside the cube with a side length of $2\times 3.54 ~{\text{\AA} }^{-1}$. (**C**) The $\omega$ dependence of $S_{xx}({{\varvec{Q}}}_1,\omega )$. The dashed line in (**B**) is the guide for ${{\varvec{q}}}={{\varvec{Q}}}_1$.

### Dynamical structure factor of magnetism

The dynamical magnetic structure factor is defined as $S_{\alpha \beta }({{\varvec{q}}},\omega )\equiv -\frac{1}{\pi }{\mathrm{Im}}G_{\alpha \beta }({{\varvec{q}}},\omega )$^22^, where $G_{\alpha \beta }({{\varvec{q}}},\omega )=\frac{1}{N}\sum _{i,j}e^{i{{\varvec{q}}}\cdot ({{\varvec{r}}}_i-{{\varvec{r}}}_j)}G^{\alpha \beta }_{ij}(\omega )$ with

3 $$\begin{aligned} G^{\alpha \beta }_{ij}(\omega )=\langle {\mathrm{GS}}|S_{i\alpha }\frac{1}{\omega +E_0-H+i\eta }S_{j\beta }|{\mathrm{GS}}\rangle . \end{aligned}$$

Here, $|{\mathrm{GS}}\rangle$ is the ground state satisfying $\alpha _i|{\mathrm{GS}}\rangle =0$ and $E_0$ is the ground-state energy. We set $\eta =10^{-6}$.

The result of $S_{xx}({{\varvec{q}}},\omega )$ for ${{\varvec{q}}}$ along the $d_2^{*e}$ line in the $q_z$-$q_x$ plane is shown in Fig. 3A. The spectra appear at $\omega /(J_1S)=0$ (see Fig. 3B) with strong intensity of $\sim O(10^{8})$ and also appear above the energy gap $\Delta$ with intensity of $\sim O(10^4)$. The energy gap in the excitation spectra $\Delta$ reflects the magnetic anisotropy arising from the CEF. For $\omega /(J_1S)>\Delta$, the large intensity appears at the energy $\omega _{90}/(J_1S)= 22.90$, where the highest peak appears at the $\Gamma$ point. At $\omega =0$, i.e., elastic energy, the maximum peak appears at ${{\varvec{q}}}={{\varvec{Q}}}_1$, as shown in Fig. 3B indicated by the dashed line. In the $\omega$ dependence of $S_{xx}({{\varvec{q}}},\omega )$, spiky peak structures appear as shown in Fig. 3C for ${{\varvec{q}}}={{\varvec{Q}}}_1$. These results indicate that the peak $S_{xx}({{\varvec{Q}}}_1)$ is governed by the elastic contribution $S_{xx}({{\varvec{Q}}}_1,0)$, which is understandable from the sum rule with respect to $\omega$ as $S_{xx}({{\varvec{Q}}}_1)=\frac{1}{2\pi }\int d\omega S_{xx}({{\varvec{Q}}}_1,\omega )$.

**Figure 4:**
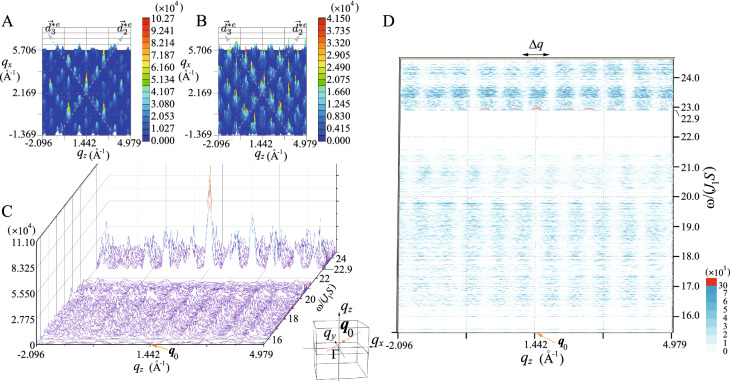
Dynamical structure factor $S_{xx}({{\varvec{q}}},\omega )$ in the $q_z$-$q_x$ plane with $q_y=3.436~{\text{\AA} }^{-1}$ for (**A**) $\omega /(J_1S)=22.90$ and (**B**) 23.07. The dashed lines indicate the $d_{2}^{*e}$ line and $d_3^{*e}$ line. (**C**) $S_{xx}({{\varvec{q}}}, \omega )$ for ${{\varvec{q}}}$ along the $d_{2}^{*e}$ line through ${{\varvec{q}}}_0=( 2.169, 3.436, 1.442)~{\text{\AA} }^{-1}$. Inset illustrates the $d_{2}^{*e}$ line through ${{\varvec{q}}}_0$ inside the cube with a side length of $8.31\times 2~{\text{\AA} }^{-1}$. (**D**) Top view of (**C**).

This is in sharp contrast to the result recently reported in the uniform long-range order of the ferrimagnetic state in the icosahedral QC^31^. Namely, the high-intensity peak appears at the ordered vector ${{\varvec{q}}}={{\varvec{0}}}$ and the lowest CEF excitation energy $\omega /(|J_1|S)=\Delta$, from which the high-intensity peaks are continuously formed in the dynamical structure factor, giving rise to the pseudo-magnon mode^31^.

Then we search the ${{\varvec{q}}}$ dependence of $S_{xx}({{\varvec{q}}},\omega )$ for $\omega =\omega _{90}$ where the large intensities appear as shown in Fig. 3A. Consequently, we identify that the maximum is located at ${{\varvec{q}}}_0\equiv ( 2.169, 3.436, 1.442)~{\text{\AA} }^{-1}$. Around ${{\varvec{q}}}={{\varvec{q}}}_0$, we find that a series of the packet structures appears along the pseudo 5-fold axis direction, as shown in Fig. 4A where the $d_{2}^{*e}$ line and $d_3^{*e}$ line through ${{\varvec{q}}}={{\varvec{q}}}_0$ is illustrated by the dashed line in the $q_z$-$q_x$ plane with $q_y= 3.436~{\text{\AA} }^{-1}$. The peak in the central packet gives the maximum $S_{xx}({{\varvec{q}}}_0,\omega _{90})= 1.027\times 10^5$. A series of packet structures with sub-leading intensity is also aligned along the pseudo 5-fold axis direction. For slightly larger $\omega$ than $\omega _{ 90}$, the packets still appear along the $d_{ 2}^{*e}$ line at slightly different positions as shown in Fig. 4B, which suggests the magnetic excitation propagating along the pseudo 5-fold direction.

Figure 4C shows $S_{xx}({{\varvec{q}}},\omega )$ for ${{\varvec{q}}}$ along the $d_{ 2}^{*e}$ line through ${{\varvec{q}}}={{\varvec{q}}}_0$. A series of the packet structures remarkably appears at the lower edge $\omega _{ 90}$ with strong intensity, which continuously forms the streak with fine structure down to the lower-$\omega$ region as also seen in the intensity plot in Fig. 4D.

Interestingly, we find that a series of the packet structures is the reflection of the bottom of the continuous mode periodic along the $d_{ 2}^{*e}$ line in the ${{\varvec{q}}}$-$\omega$ plane as shown in Fig. 4D. The period of the streak structure is evaluated as $\Delta {q}\sim 0.6~{\text{\AA} }^{-1}$ in the reciprocal space. From the relation of the wavenumber and wavelength $\Delta {q}=2\pi /\lambda$, the scale of the wavelength is estimated to be $\lambda \sim 10~{\text{\AA} }$. It turns out that this corresponds to the diameter of the IC $d=10.56~{\text{\AA} }$ (see Fig. 1A). Since the hedgehog is the magnetic texture on the IC, the excitation gives rise to the dynamics whose intensity decreases with periodicity $\Delta {q}\sim 2\pi /d$ with distance from ${{\varvec{q}}}_0$ in the reciprocal space of the QC. A series of the packet structure as well as the intensity streak in the ${{\varvec{q}}}$-$\omega$ plane also appears along the $d_3^{*e}$ direction (Supplementary information, Fig. S3). The emergence of the intensity streak with fine structure in the ${{\varvec{q}}}$-$\omega$ plane indicates unique excitation mode along the 5-fold axis direction, which is considered to be characteristic of the hedgehog in the icosahedral QC.

**Figure 5:**
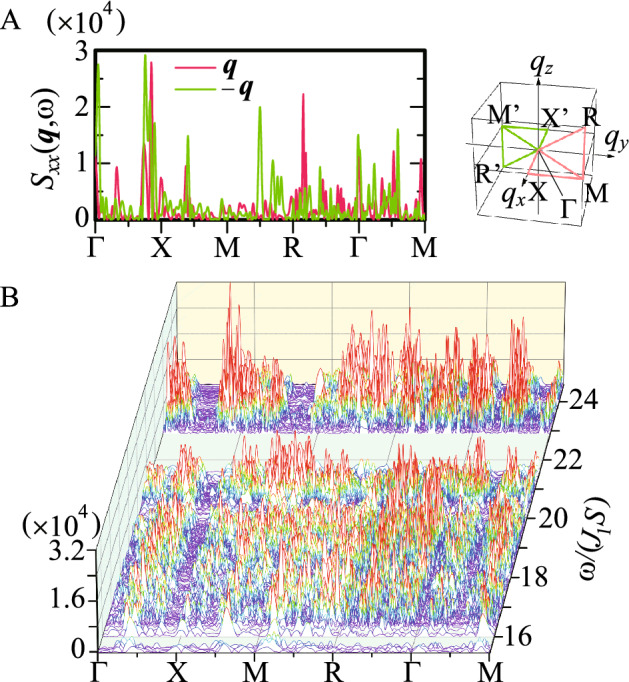
(**A**) Dynamical structure factor $S_{xx}({{\varvec{q}}},\omega )$ $[ S_{xx}({-{\varvec{q}}},\omega )]$ at $\omega /(J_1S)=23.18$ for ${{\varvec{q}}}$ along the pink (green) lines in the inset. Inset illustrates the cube with a side length of $2.56\times 2~{\text{\AA} }^{-1}$. (**B**) $|S_{xx}({{\varvec{q}}},\omega )-S_{xx}({-{\varvec{q}}},\omega )|$ for ${{\varvec{q}}}$ along the pink lines in the inset and for $\omega _{N}\le \omega \le \omega _{1}$.

### Non-reciprocal magnetic excitation in QC

To further clarify the general property of the dynamics of the hedgehog state, we show $S_{xx}({{\varvec{q}}},\omega )$ at $\omega /(J_1S)=23.18$ for ${{\varvec{q}}}$ along the pink lines in the cube whose side is parallel to the 2-fold axis $q_{\alpha }\in [0,2.56]~{\text{\AA} }^{-1}$ $(\alpha =x,y,$ and *z*) in the inset of Fig. 5A. Here we also plot $S_{xx}(-{{\varvec{q}}},\omega )$ along the green line in the inset of Fig. 5A. We see remarkable differences in the intensity for ${{\varvec{q}}}$ and $-{{\varvec{q}}}$. In Fig. 5B, we plot $|S_{xx}({{\varvec{q}}},\omega )-S_{xx}({-{\varvec{q}}},\omega )|$ for ${{\varvec{q}}}$ along the pink lines in the inset of Fig. 5A. The finite values indicate that $S_{xx}({{\varvec{q}}},\omega )\ne S_{xx}(-{{\varvec{q}}},\omega )$. These results indicate that non-reciprocal magnetic excitation appears in the hedgehog state in the QC. This is, to our best knowledge, the first discovery of the non-reciprocal magnetic excitation in the topological magnetic long-range order in QC.

We confirmed that non-reciprocal magnetic excitation does not appear in the case of the collinear magnetic order in the QC (Supplementary information, Fig. S4). This implies that the noncollinear and noncoplanar magnetic structure on the IC of the hedgehog (Fig. 1A) is the origin of the nonreciprocal excitation. Recently, non-reciprocal magnetic excitation from the uniform ferrimagnetic order (characterized by the zero topological charge $n_{\mathrm{TC}}=0$) in the icosahedral QC has been shown to appear^31^. These results suggest that non-reciprocal excitation is common character of the noncollinear and noncoplanar alignment of the magnetic moments on the IC. As shown in Fig. 5B, emergence of many spiky peaks with fine structure as continuum are the consequence of the QC structure, which is in sharp contrast to the magnon branch in periodic crystals as the collective mode. This gives rise to the emergence of nonreciprocity as continuum in the vast extent of the ${{\varvec{q}}}$-$\omega$ plane (see Fig. 5B), whose feature is unique to the QC.

### Summary and discussion

We have revealed the dynamical as well as static property of the hedgehog state in the QC. The FM hedgehog state is shown to be characterized by the triple-*Q* state. The magnetic dynamical structure factor shows highly structured energy and momentum dependences unique to the QC. We have discovered the magnetic excitation mode along the pseudo 5-fold axis direction. A series of the packet structure in the dynamical structure factor is found to exist, which is shown to be the reflection of the periodic streak structure in the reciprocal lattice ${{\varvec{q}}}$-energy $\omega$ plane. Non-collinear and non-coplanar magnetic alignment of the hedgehog state gives rise to non-reciprocal magnetic excitations which appear in the vast extent of the energy and momentum plane.

In the uniform long-range order of the ferrimagnetic state, the high-intensity peaks appear continuously from the ordered vector ${{\varvec{q}}}=\mathbf{0}$ and the lowest CEF excitation energy $\omega =\Delta |J_1|S$, which are identified as the pseudo-magnon mode^31^. On the contrary, in the dynamical structure factor for the uniform hedgehog order, the high-intensity peaks do not appear at the ordered vector ${{\varvec{Q}}}_i$ for $i=1$, 2, and 3 beyond the CEF excitation energy. This implies that the peak in the static structure factor at the triple *Q* vector ${{\varvec{q}}}={{\varvec{Q}}}_i$ is governed by the elastic $(\omega =0)$ contribution of the dynamical structure factor for the uniform hedgehog order.

The streak structure with periodicity characterized by the wavelength corresponding to the diameter of the IC in the ${{\varvec{q}}}$-$\omega$ plane is considered to be the unique character of the excitation from the uniform hedgehog order. To establish this point, the systematic analysis of the dynamical structure factor in the magnetically ordered states in the icosahedral QC is necessary, which is left for future studies.

The non-reciprocal magnetic excitation has also been found to emerge in the uniform ferrimagnetic order^31^. Hence, as noted above, non-reciprocity is considered to be general feature of the excitation from the non-collinear and non-coplanar magnetic texture on the IC.

Our results are useful not only for resolving the magnetic structure of the long-range order discovered recently in Tb-based icosahedral QC, but also for future neutron measurements of the magnetic dynamics in the QC. So far, the dynamical structure factor in the magnetically ordered phase in the QC has not been reported. It is expected that present study stimulates future experiments to detect the dynamical property in the QC and also in the approximant crystal.

## Methods

### Theory of Magnetic Excitation in QC

Magnetic excitation from the uniform hedgehog long-range order in the QC can be calculated by transforming spin operators in the model (1) into boson operators. Since the hedgehog is a noncoplanar magnetic state, it is convenient to introduce the local coordinate at each Tb site^32^. The unit vectors in the global *xyz* coordinate $\hat{r}_1=\hat{x}$, $\hat{r}_2=\hat{y}$, and $\hat{r}_3=\hat{z}$ are expressed by the local orthogonal coordinate with the unit vector $\hat{{\varvec{e}}}^i_{3}$, whose direction is indicated by the polar angles $(\theta _i, \phi _i)$, as

4 $$\begin{aligned} \hat{{\varvec{r}}}_{\alpha }=R_{\alpha \beta }^{i}\hat{{\varvec{e}}}_{\beta }^{i} \end{aligned}$$

(see Fig. 1C). Here, $R^{i}$ is the rotation matrix defined as

5 $$\begin{aligned} R^{i}= \begin{bmatrix} \cos \theta _i\cos \phi _i &{} -\sin \phi _i &{} \sin \theta _i\cos \phi _i \\ \cos \theta _i\sin \phi _i &{} \cos \phi _i &{} \sin \theta _i\sin \phi _i \\ -\sin \theta _i &{} 0 &{} \cos \theta _i \end{bmatrix}. \end{aligned}$$

Then, the first term in Eq. (1) is expressed as

6 $$\begin{aligned} \sum _{\langle i,j\rangle }J_{i,j}({{\varvec{S}}}_i\cdot {{\varvec{e}}}_{\alpha }^i)({{\varvec{S}}}_j\cdot {{\varvec{e}}}_{\beta }^j)\sum _{\gamma }R_{\alpha ,\gamma }^{i}R_{\gamma ,\beta }^{j}. \end{aligned}$$

By using ${{\varvec{S}}}_i\cdot \hat{{\varvec{e}}}_1^i=(S_i^{+}+S_i^{-})/2$ and ${{\varvec{S}}}_i\cdot \hat{{\varvec{e}}}_2^i=(S_i^{+}-S_i^{-})/(2i)$ where $S_i^{+}$ and $S_i^{-}$ are raising and lowering “spin” operators, respectively, we apply the Holstein-Primakoff transformation^28^ to *H*. Namely, “spin” operators are expressed by the boson operators as $S_i^{+}=\sqrt{2S-n_i}a_i$, $S_i^{-}=a_i^{\dagger }\sqrt{2S-n_i}$ and ${{\varvec{S}}}_i\cdot \hat{{\varvec{e}}}_3^i=S-n_i$ with $n_i\equiv a_i^{\dagger }a_i$. We retain the quadratic terms with respect to $a_i^{\dagger }$ and $a_i$, which are considered to be at least valid for the ground state. In the noncollinear magnetic state as the hedgehog, anomalous terms such as $a_i^{\dagger }a_j^{\dagger }$ and $a_ia_j$ appear. The resultant *H* is expressed as

7 $$\begin{aligned} H=[\chi ^{\dagger } \tilde{\chi }]\Lambda \begin{bmatrix} \chi \\ \tilde{\chi }^{\dagger } \end{bmatrix}, \end{aligned}$$

where $\chi ^{\dagger }=(a^{\dagger }_1,a^{\dagger }_2,\cdots ,a^{\dagger }_N)$ and $\Lambda$ is the $2N\times 2N$ matrix. By performing the para unitary transformation

8 $$\begin{aligned} \begin{bmatrix} \zeta \\ \tilde{\zeta }^{\dagger } \end{bmatrix} =\mathcal{J} \begin{bmatrix} \chi \\ \tilde{\chi }^{\dagger } \end{bmatrix} \end{aligned}$$

where $\zeta =(\alpha _1, \alpha _2, \cdots , \alpha _N)$ and $\mathcal{J}$ is the para unitary matrix^33^, we obtain

9 $$\begin{aligned} H=[\zeta ^{\dagger } \tilde{\zeta }] \begin{bmatrix} \bar{\omega } &{} \bar{0} \\ \bar{0} &{} \tilde{\omega } \end{bmatrix} \begin{bmatrix} \zeta \\ \tilde{\zeta }^{\dagger } \end{bmatrix}. \end{aligned}$$

Here, $\bar{\omega }$ is the $N\times N$ diagonal matrix $\bar{\omega }={\mathrm{diag}}(\omega _1, \omega _2, \cdots , \omega _N)$ with $\omega _i>0$, $\tilde{\omega }={\mathrm{diag}}(\omega _N, \omega _{N-1}, \cdots , \omega _1)$, and $\bar{0}$ is the $N\times N$ matrix with all elements being zero. Here, the index *i* represents the eigenvalue of the excitation energy from the magnetically ordered state.

## Supplementary Information

Supplementary Information.

## Data Availability

All the data supporting the findings are available from the corresponding author upon reasonable request.
