# Supplementary material for: Magnetic dynamics of hedgehog in icosahedral quasicrystal
Source: Sci Rep. 2022 Sep 15;12:15514. doi: 10.1038/s41598-022-19870-6 (PMC9478144; doi:10.1038/s41598-022-19870-6)
Supplement: Supplementary file 1 — Supplementary Information. [file 41598_2022_19870_MOESM1_ESM.pdf]

Supplementary Information for
“ Magnetic dynamics of hedgehog in icosahedral quasicrystal ”

Shinji Watanabe*

Department of Basic Sciences, Kyushu Institute of Technology,

Kitakyushu, Fukuoka 804-8550, Japan

(Dated: June 17, 2022)

I. Minimal model applied to the quasicrystal

In the main text, the minimal model (1) is applied to the quasicrystal (QC), where the nearest-neighbor (N.N.) interaction J_1 and the next-nearest-neighbor (N.N.N.) interaction J_2 operate on the magnetic moments at the Tb sites not only as the intra-icosahedron (IC) interaction but also as the inter-IC interaction. In Fig. S1, the N.N. interaction J_1 and the N.N.N. interaction J_2 for the intra IC and inter IC are illustrated as the solid line and the dashed line, respectively. The inter-IC interaction operates on each neighboring pair of the ICs located at the 30 vertices of the icosidodecahedron (the number of the neighboring pair of the ICs is 60), which are connected by the green (brown) line in Fig. 1*B* in the main text. On the intra-IC interaction, the magnetic moment at each Tb site located at the 12 vertices of the IC interacts with the magnetic moment at the five N.N. Tb sites by J_1 and also interacts with that at the five N.N.N. Tb sites by J_2.

In ref. [1], it has been shown that the hedgehog state in the IC displayed in Fig. 1*A* forms the uniform arrangement in the QC as the ground state as shown in Fig. 1*B*. The formation

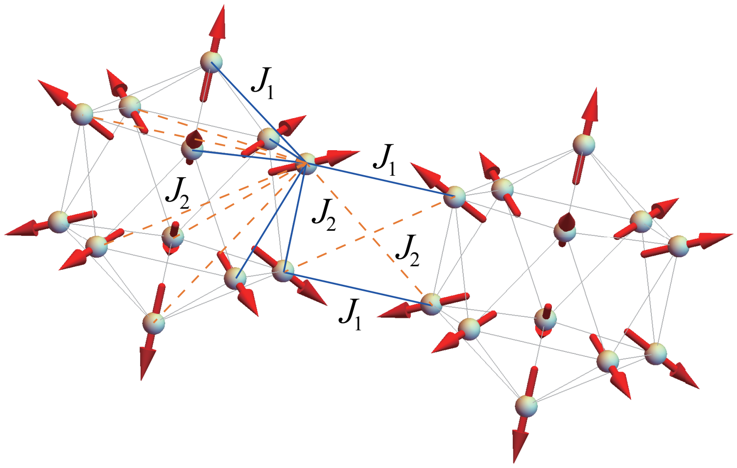

FIG. S1. (color online) Nearest neighbor ICs in the QC. Nearest neighbor interaction J_1 and next nearest neighbor interaction J_2 for intra IC and inter IC are illustrated as the solid line and dashed line, respectively.

of the long-range order of the hedgehog is intuitively understood as follows. If the hedgehog is uniformly distributed in the QC, the magnetic moments pointing outward from the IC interact antiferromagnetically with the neighboring IC as shown in Fig. S1 at least for the N.N. and N.N.N. interactions. We confirmed that the magnetic moments on the N.N.N. pairs for all the neighboring ICs in the QC are aligned in the antiparallel direction each other (see Fig. S1). In addition, we also confirmed that the magnetic moments on the N.N. pairs for all the neighboring ICs in the QC are also aligned antiferromagnetically, i..e, $\langle \boldsymbol{S}_i \cdot \boldsymbol{S}_j \rangle < 0$. This implies that if the N.N. and N.N.N. AFM interactions operate on the hedgehog as the inter-IC interaction, the hedgehog is stabilized in the IC to IC. Consequently, uniform arrangement of the hedgehog is realized in the cluster shown in Fig. 1B and also even in the outer clusters in the self-similar manner, giving rise to the uniform long-range order in the QC. This is actually the case of the model (1) applied to the $Cd_{5.7}Yb$-type QC with the AFM interactions $J_2/J_1 > 2$, as discussed in ref. [1]. In the main text, the magnetic dynamical as well as static structure factor is calculated for this parameter region in the cluster with $N = 360$ Tb sites shown in Fig. 1B.

II. Magnetic structure factor

In Fig. 2B in the main text, the magnetic structure factor $F_s(\boldsymbol{q})$ shows the largest peak at $\boldsymbol{Q}_1 \equiv (1.77, 0, 1.02)$ Å^{-1}. By performing the numerical calculation, we have confirmed that the largest peak in $F_s(\boldsymbol{q})$ also appears at $\boldsymbol{Q}_2 \equiv (0, 1.02, 1.77)$ Å^{-1} as shown in Fig. S2A and at $\boldsymbol{Q}_3 \equiv (1.02, 1.77, 0)$ Å^{-1} as shown in Fig. S2B. Namely, $F_s(\boldsymbol{Q}_1) = F_s(\boldsymbol{Q}_2) = F_s(\boldsymbol{Q}_3)$ holds. This implies that the hedgehog state is characterized by the triple-Q ($\boldsymbol{Q}_1$, $\boldsymbol{Q}_2$, and $\boldsymbol{Q}_3$) state. The results shown in Figs. S2A and S2B confirm the symmetry argument for the triple-Q state in the main text.

III. Dynamical structure factor

In the main text, the dynamical structure factor $S_{xx}(\boldsymbol{q}, \omega)$ for $\boldsymbol{q}$ along the d_2^{*e} line through $\boldsymbol{q}_0$ is shown in Figs. 4C and 4D. It is noted that as shown in Figs. 4A and 4B, a series of the packet structures appears in the pseudo 5-fold direction, i..e, not only along the d_2^{*e} direction but also along the d_3^{*e} direction. Here, we show the results of the dynamical structure factor along the d_3^{*e} direction. Figure S3A shows $S_{xx}(\boldsymbol{q}, \omega)$ for $\boldsymbol{q}$ along the d_3^{*e} line through $\boldsymbol{q}_0$. At $\omega_{90}/(J_1 S) = 22.9$, the maximum of $S_{xx}(\boldsymbol{q}, \omega_{90})$ appears at $\boldsymbol{q} = \boldsymbol{q}_0 \equiv (2.169, 3.436, 1.442)$ Å^{-1}

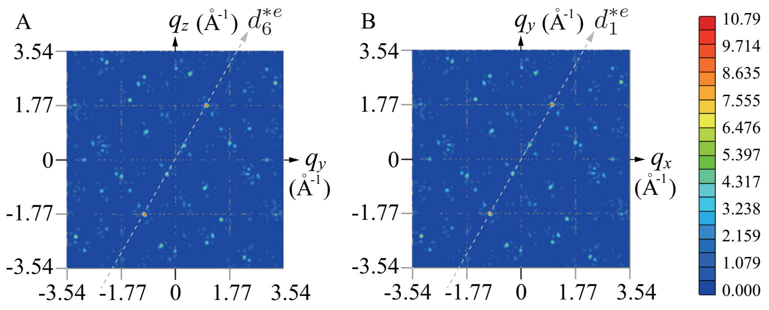

FIG. S2. (color online) Top view of $F_s(\boldsymbol{q})$ in the q_y-q_z plane for $q_x = 0$ (A) and in the q_x-q_y plane for $q_z = 0$ (B). The gray dashed line in (A) and (B) denotes the pseudo 5-fold axis d_6^{*e} and d_1^{*e}, respectively, defined in Fig. 2D in the main text.

where the largest sharp peak appears. It is remarkable that at $\omega/(J_1 S) = 22.9$, a series of the packet structures appears around $\boldsymbol{q} = \boldsymbol{q}_0$.

Interestingly, streak composed of fine structures from each packet structure continues down to the lower-ω region in Fig. S3A. This is clearly seen in the top view of $S_{xx}(\boldsymbol{q}, \omega)$ plotted in Fig. S3B. The $\boldsymbol{q}$-ω plane is streaked with fine structures, which also continues even above $\omega/(J_1 S) = 22.9$ with larger intensities. The period of the streak structure in the reciprocal space is evaluated as $\Delta q \sim 0.6$ Å^{-1}. From the relation of the wavenumber and the wavelength $\Delta q = 2\pi/\lambda$, the wavelength λ is estimated to be $\lambda \sim 10$ Å. It turns out that this length scale corresponds to the diameter of the IC $d = 10.56$ Å[see Figs. 1A and 1B in the main text]. Since the hedgehog is the magnetic texture on the IC, the Fourier transform of the magnetic dynamical correlation $G_{ij}^{\alpha\beta}(\omega)$ [see Eq. (9) in the main text] in the QC gives rise to the intensity decreasing with periodicity $\Delta q \sim 2\pi/d$ with distance from $\boldsymbol{q}_0$ in the reciprocal space.

IV. Collinear ferromagnetic order in icosahedral quasicrystal

In the main text, we have shown that in the QC, the non-reciprocal magnetic excitation appears in the noncollinear magnetic structure of the hedgehog on the IC in Fig. 4. In this section, we discuss the magnetic excitation in the collinear magnetic order in the QC. We apply the model (1) in the main text to the $Cd_{5.7}Yb$-type QC [see Fig. 1B] with the N.N. interaction J_1 and the N.N.N. interaction J_2 as the intra-IC and inter-IC interactions [see Fig. S1]. Here, we consider the case that the uniaxial anisotropy is along the z direction for all the sites as shown in Fig. S4A. When the ferromagnetic (FM) interaction works

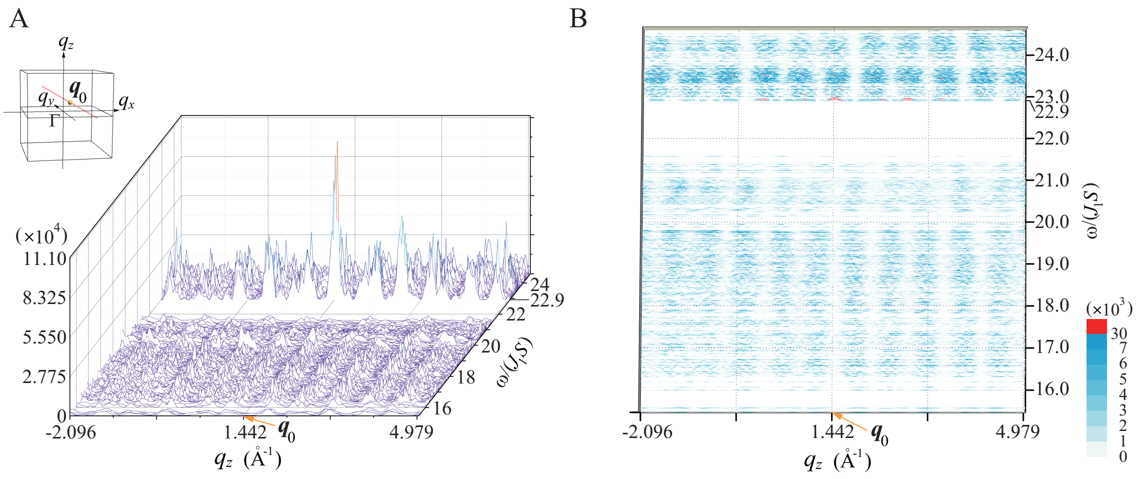

FIG. S3. (color online) (A) Dynamical structure factor $S_{xx}(\boldsymbol{q}, \omega)$ for $\boldsymbol{q}$ along the d_3^{*e} line through $\boldsymbol{q}_0 = (2.169, 3.436, 1.442)$ Å^{-1} in the reciprocal-lattice space. Inset illustrates the d_3^{*e} line through $\boldsymbol{q}_0$ inside the cube with a side length of 8.31×2 Å^{-1}. (B) Top view of A for $\omega_N \le \omega \le \omega_1$.

dominantly between the magnetic moments at the N.N. sites each other, the collinear FM long-range order is realized in the QC, as shown in Fig. S4B.

Then, by using the method described in the main text, we have calculated the dynamical structure factor for $J_1 = -1.0$, $J_2 = 0.0$, and $D = 30$ with $S = 6$ in the $N = 360$ lattice of the QC. Figure S4C shows the result of $S_{xx}(\boldsymbol{q}, \omega)$ (solid line) at $\omega/(|J_1|S) = 27.28$, which is the lowest excitation energy, for $\boldsymbol{q}$ along the pink lines in the inset of Fig. S4C. We also plot $S_{xx}(-\boldsymbol{q}, \omega)$ along the green line in the inset of Fig. S4C as the dashed line. The results show that both the data coincide. Furthermore, for comparison with Fig. 5B in the main text, we plot $|S_{xx}(\boldsymbol{q}, \omega) - S_{xx}(-\boldsymbol{q}, \omega)|$ for $\boldsymbol{q}$ along the pink line in the inset of Fig. S4C for $27.28 \le \omega/(|J_1|S) \le 36.19$ in Fig. S4D. The intensity expressed by each purple line located at ω_i for $i = 1, \cdots, N$ on the $\boldsymbol{q}$-ω plane [ω_i is the eigenvalue in Eq. (7) in the main text, e.g., $\omega_1/(|J_1 S|) = 27.28$ and $\omega_N/(|J_1|S) = 36.19$] indicates zero value everywhere, which implies that $S_{xx}(\boldsymbol{q}, \omega) = S_{xx}(-\boldsymbol{q}, \omega)$ holds. Namely, non-reciprocal magnetic excitation does not appear in the collinear FM long-range order in the QC. This is in sharp contrast to the results shown in Figs. 5A and 5B in the main text. These results indicate that noncollinear magnetic structure on the IC as the hedgehog is the origin of emergence of the non-reciprocal magnetic excitation.

Finally, the relation to real compounds is remarked. The collinear FM state shown in Fig. S4B is unlikely to be realized in the QC composed of the rare-earth ion such as Tb^{3+}

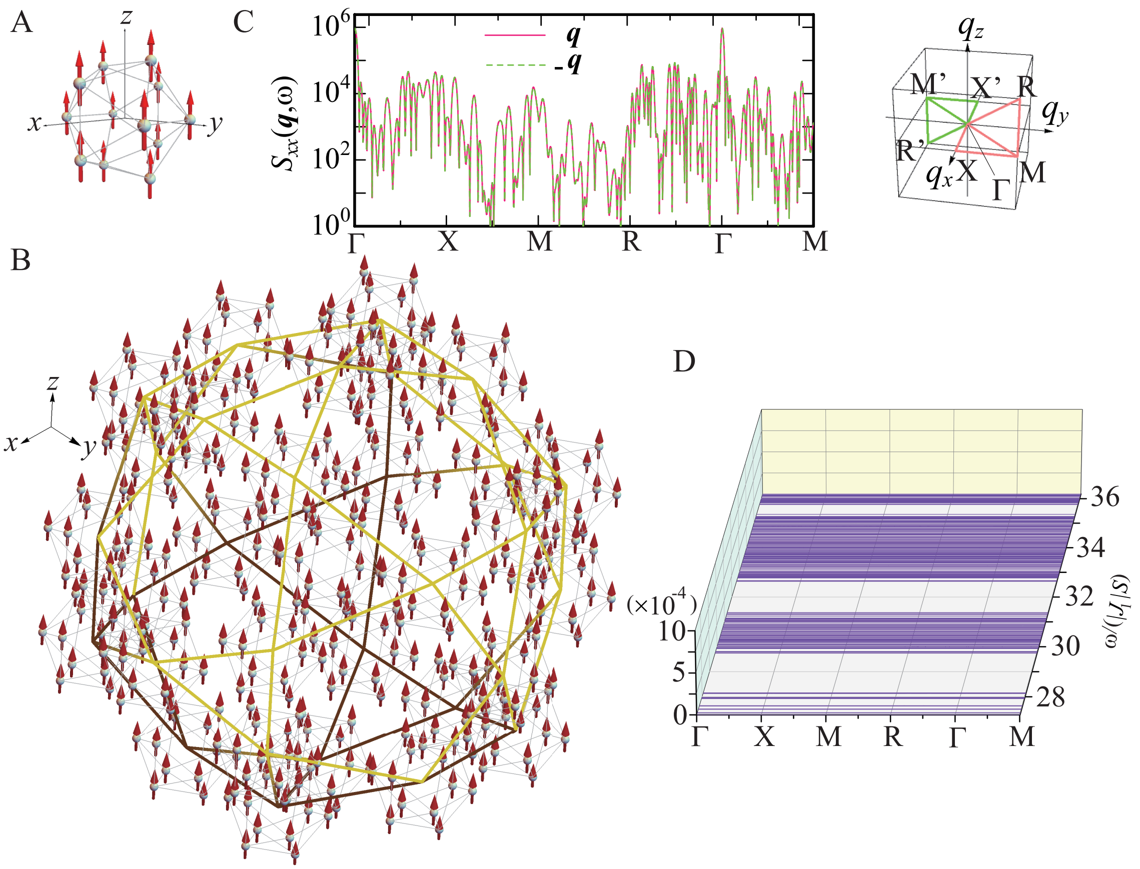

FIG. S4. (color online) (A) Collinear FM state on the IC. (B) Collinear FM long-range order in the QC. Green (brown) lines at the front (back) connect the vertices of the icosidodecahedron. (C) Dynamical structure factor $S_{xx}(\boldsymbol{q},\omega)$ (solid line) at $\omega/(|J_1|S) = 27.28$ for $\boldsymbol{q}$ along the pink line in the inset. $S_{xx}(-\boldsymbol{q},\omega)$ (dashed line) is also plotted for $\boldsymbol{q}$ along the green line in the inset. Inset illustrates the cube with a side length of 2.56×2 Å^{-1} in the reciprocal-lattice space. (D) $|S_{xx}(\boldsymbol{q},\omega) - S_{xx}(-\boldsymbol{q},\omega)|$ for $\boldsymbol{q}$ along the pink line in the inset of (C).

with finite orbital angular momentum $L \neq 0$ because of the CEF effect at each site, but is possible to be realized in the systems composed of magnetic ions such as Gd^{3+} and Eu^{2+} with $L = 0$. Such a state is considered to correspond to the case for $D = 0$ in the model (1). In this section, we have discussed the collinear FM state by setting $D = 30$ in the model (1) for comparison with the results in Fig. 5 in the main text. The collinear FM state for $D = 0$ in the QC will be discussed in the separate paper elsewhere.

[1] S. Watanabe, Magnetism and topology in Tb-based icosahedral quasicrystal, *Sci. Rep.* **11**, 17679 (2021).

[2] T. Hiroto *et al.*, Noncoplanar ferrimagnetism and local crystalline-electric-field anisotropy in the quasicrystal approximant $Au_{70}Si_{17}Tb_{13}$, *J. Phys.: Condens. Matter* **32**, 415802 (2020).

[3] T. Holstein and H. Primakoff, Field Dependence of the Intrinsic Domain Magnetization of a Ferromagnet, *Phys. Rev.* **58**, 1098 (1940).
